# A Heart Rate Matched Patch for Mechano-Chemical Treatment of Myocardial Infarction: Optimal Design and Transspecies Application

**DOI:** 10.34133/research.0517

**Published:** 2024-11-22

**Authors:** Yuanbo Jia, Zhao Wei, Jinteng Feng, Meng Lei, Yanshen Yang, Jingyi Liu, Yufei Ma, Weiguo Chen, Guoyou Huang, Guy M. Genin, Xiaogang Guo, Yan Li, Feng Xu

**Affiliations:** ^1^ Department of Hepatobiliary Surgery and Liver Transplantation, The Second Affiliated Hospital of Xi’an Jiaotong University, Xi’an 710004, P.R. China.; ^2^ Key Laboratory of Surgical Critical Care and Life Support (Xi’an Jiaotong University), Ministry of Education, Xi’an, P.R. China.; ^3^ Bioinspired Engineering and Biomechanics Center (BEBC), Xi’an Jiaotong University, Xi’an 710049, P.R. China.; ^4^ MOE Key Laboratory of Biomedical Information Engineering, School of Life Science and Technology, Xi’an Jiaotong University, Xi’an 710049, P.R. China.; ^5^ Department of Thoracic Surgery, The First Affiliated Hospital of Xi’an Jiaotong University, Xi’an 710061, P.R. China.; ^6^ Department of Cardiology, Tangdu Hospital, the Air Force Military Medical University, Xi’an, Shaanxi 710038, P.R. China.; ^7^ Department of Engineering Mechanics, School of Civil Engineering, Wuhan University, Wuhan 430072, P.R. China.; ^8^ Department of Mechanical Engineering & Materials Science, Washington University in St. Louis, St. Louis, MO 63130, USA.; ^9^ NSF Science and Technology Center for Engineering Mechanobiology, Washington University in St. Louis, St. Louis, MO 63130, USA.; ^10^ Department of Cardiology, the First Affiliated Hospital, School of Medicine, Zhejiang University, Hangzhou 310003, P.R. China.

## Abstract

After myocardial infarction (MI), ventricular dilation and the microscopic passive stretching of the infarcted border zone is the meaning contributor to the continuous expansion of myocardial fibrosis. Epicardial hydrogel patches have been demonstrated to alleviate this sequela of MI in small-animal models. However, these have not been successfully translated to humans or even large animals, in part because of challenges in attaining both the greater stiffness and slower viscoelastic relaxation that mathematical models predict to be optimal for application to larger, slower-beating hearts. Here, using borate-based dynamic covalent chemistry, we develop an injectable “heart rate matched” viscoelastic gelatin (VGtn) hydrogel with a gel point tunable across the stiffnesses and frequencies that are predicted to transspecies and cross-scale cardiac repair after MI. Small-animal experiments demonstrated that, compared to heart rate mismatched patches, the heart rate matched VGtn patches inhibited ventricular bulging and attenuated stress concentrations in the myocardium after MI. In particular, the viscoelastic patch can coordinate the microscopic strain at the infarction boundary. VGtn loaded with anti-fibrotic agents further reduced myocardial damage and promoted angiogenesis in the myocardium. The tuned heart rate matched patches demonstrated similar benefits in a larger-scale and lower heart rate porcine MI model. Results suggest that heart rate matched VGtn patches may hold potential for clinical translation.

## Introduction

Revascularization (e.g., coronary artery bypass grafting and coronary artery stenting) has been clinically successful in saving thousands of patients with myocardial infarction (MI) [1]. However, acute inflammation and irreversible myocardial injury due to ischemia are inevitable because the procedure cannot be performed immediately after MI. In particular, mechanical incoordination caused by contractile insufficiency of the fibrotic scar not only impairs myocardial ejection function but also plays an important role in the expansion of myocardial fibrosis. The scar tissue cannot contract, stretching passively when the surrounding healthy tissue contracts during systole. The resulting passive stretch, especially for infarcted regions in the left ventricle (LV), can trigger pathological tissue remodeling that leads to dilatation of the ventricle, thinning of the scar, and eventually congestive heart failure or ventricular rupture [2,3]. Therefore, additional therapeutic approaches are desired to reduce post-ischemic myocardial inflammation to reduce myocardial necrosis and to harmonize myocardial mechanics to enhance ventricular ejection and prevent ventricular dilatation and progressive fibrotic cardiomyopathy [4–6].

**Fig. 1.**
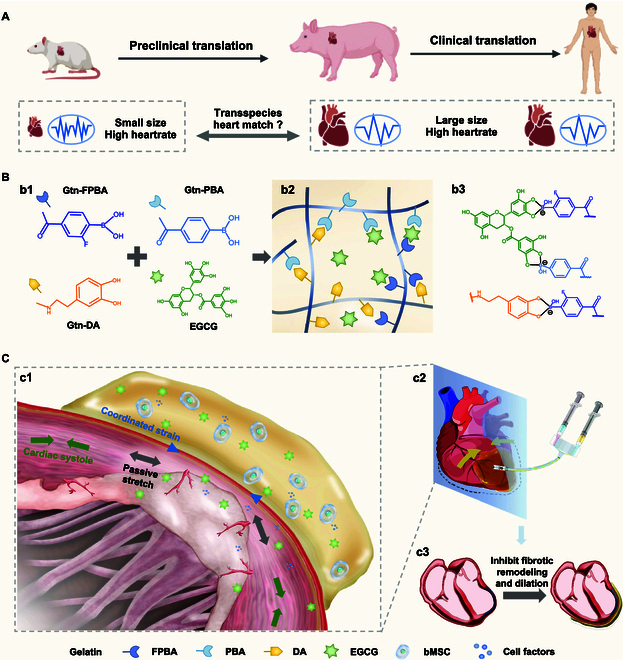
A multifunctional hydrogel patch with tunable viscoelastic networks for treatment of cardiac fibrosis. (A) Differences of heart size and heart rate in transspecies translation. (B) Synthesis and chemical structures of the VGtn hydrogel. (1) Precursors of the hydrogels. (2) Schematic of the hydrogel network. (3) Dynamic boronate bonds of PBA-DA, PBA-EGCG, and FPBA-EGCG. (C) Schematic of MI treatment using VGtn hydrogel. (1) The hydrogel can reduce scar straining, especially on the infarction border, and can deliver therapeutic drugs and cells to reduce fibrotic remodeling. (2) Minimally invasive injection of the hydrogel using a double syringe. (3) The hydrogel forms a patch that may inhibit dilatation of fibrotic hearts after MI.

Injectable hydrogel patch, with programmable mechanical and chemical properties, are considered to be a powerful tool for harmonizing myocardial mechanics while delivering therapeutic factors, such as drugs or cell [5,7,8]. Although stiff, elastic epicardial patches prevent rupture of ventricular wall and improve function from the perspective of static structural mechanics [9–11], they may interfere with dynamic cardiac output by restricting heart ejection or locking tissue into a strained state [12–14]. To match with the heart, a dynamic organ with a certain range of contractile frequency (heart rate) patches with dynamic mechanics that are adaptable to heart rate may be more advantageous. Viscoelastic patches have been shown recently to reduce LV dilatation and improve LV ejection fraction relative to stiff and elastic patches [15]. The minimized effect of prestress in patch grafting and the balance between restraining dilatation and maintaining normal heart function is reached by constructing hydrogel with gel point frequency (*f_GP_*) that matches the dynamic “heart rate” and gel point modulus ($G_{\mathrm{GP}}^{\prime }$) that matches the static myocardial structure and stiffness. Here, the gel point frequency *f_GP_* indicates the frequency at which its storage modulus $G_{\text{patch}}^{\prime }$ equals its loss modulus $G_{\text{patch}}^{\prime \prime }$ (i.e., gel point modulus $G_{\mathrm{GP}}^{\prime }$). Although this has been demonstrated in rodent models of acute MI, there remains obstacles to transspecies clinical translation, mainly because of differences in heart rate (3 to 6 Hz for rodent, ~1 Hz for humans) and body size across species [16]. Thus, hydrogels that can independently modulate *f_GP_* and $G_{\mathrm{GP}}^{\prime }$ according to transspecies heart rate and myocardial structure hold promise for solving this challenge (Fig. 1A).

Biochemical factors also play an important role in the progression and treatment of MI. Reactive oxygen species (ROSs) trigger myocardial oxidative stress after MI that injures cell membranes and gives rise to myocyte death and subsequent inflammation [17]. Removal of ROS reduces pathological ventricular remodeling following acute MI [18]. Epigallocatechin-3-gallate (EGCG), a green tea-derived polyphenol, has been believed to be effective in reducing acute inflammation after tissue injury and prevent the fibrosis process due to its efficient scavenging of ROSs [19]. In addition, stem cell-based cell therapy has shown great potential in the treatment of MI. Although in vivo differentiation into mature myocardial tissue remains technically difficult, delivery of stem cells in the infarcted region has shown great potential for revascularization, prevention of fibrosis, etc. However, attempts to integrate anti-fibrotic agents with harmonizing myocardial mechanics are scarce, and the chemo-mechanical treatment efficiency on MI remains elusive [20,21].

To address these challenges, we designed tunable, viscoelastic gelatin (VGtn)-based hydrogel patches. By modulating dynamic boronate crosslinking kinetics, we obtained a hydrogel set with heart rate matched *f_GP_* across different species and independently tunable $G_{\mathrm{GP}}^{\prime }$. EGCG can be combined with phenylboronic acid to achieve loading and controlled release of antifibrotic drugs, while the Gtn hydrogel can serve as a good scaffold for cell delivery (Fig. 1B). We demonstrated the chemo-mechanical effect of heart rate matched hydrogel patches in preventing myocardial fibrosis and improving heart function by assessing the performance of VGtn hydrogel patches in rodent models of acute MI and advanced fibrotic cardiomyopathy. We further validated the cross-scale and transspecies translation potential of heartrate-matched patches through a preclinical porcine model (Fig. 1).

## Results

### A heart rate matched Gtn hydrogel with independently tunable gel point parameters

**Fig. 2.**
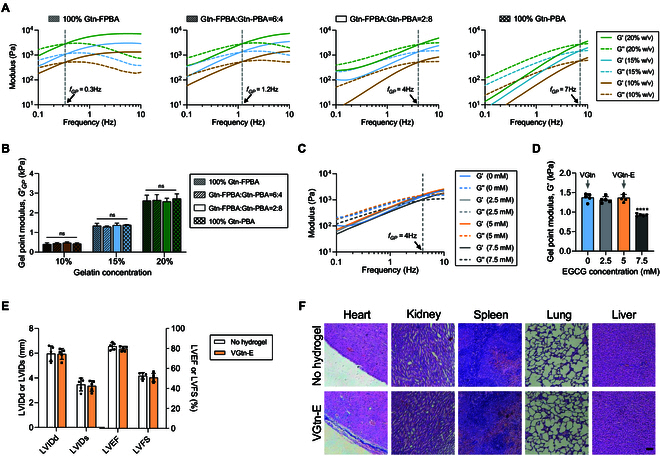
VGtn hydrogel patches have tunable gel point properties and are safe for cardiac implantation. (A) Rheological characterization of VGtn hydrogels with prescribed weight fractions and ratios of Gtn-PBA and Gtn-FPBA. The frequency of the gel point (gray dashed line) can be controlled by the Gtn-PBA:Gtn-FPBA ratio, and the gel point modulus *G*′ can be controlled independently by the weight fraction of Gtn. (B) Gel point modulus GP-*G*′ of VGtn, which varied with Gtn concentration but not the ratio of Gtn-PBA:Gtn-FPBA. (C and D) Rheological characterization of VGtn hydrogels with different EGCG concentrations. (E) Left ventricular internal diameter end diastole (LVIDd), internal diameter end systole (LVIDs), ejection fraction (LVEF), and fractional shortening (LVFS) measured in rats implanted with VGtn-E or receiving sham treatment, measured 2 months after implantation or sham treatment. (F) H&E staining of organs of healthy rats implanted with VGtn-E or receiving sham treatment, harvested 2 months after implantation or sham treatment. Scale bar, 100 μm. *n* = 5 independent samples for each group. Data are shown as mean ± SD and compared by one-way ANOVA followed by Bonferroni’s post hoc test. No significant differences (*P* > 0.05) were found in the data of (B) and (E). **** indicates *P* < 0.0001 in (D).

Gtn backbone chains were crosslinked by inserting functional domains that could be modulated to adjust $G_{\mathrm{GP}}^{\prime }$, *f_GP_*, and biodegradability and to load the hydrogel with therapeutic factors. To tune *f_GP_*, 4-carboxy-phenylboronic acid (PBA) and 4-carboxy-2-fluorophenylboronic acid (FPBA) were modified on Gtn, and then crosslinked with dopamine (DA)-grafted Gtn (Gtn-DA) to form dynamic PBA-DA and FPBA-DA bonds (Fig. 1 and Fig. S1). Because of the different affinities of PBA and FPBA to catechol on DA [22] (Fig. 2A), *f_GP_* could be tuned from that of pure FPBA-DA (0.3 Hz) to that of pure PBA-DA (~6 Hz), encompassing the ranges of both humans (0.8 to 2.2 Hz) [23,24] and rats. This could be done without changing $G_{\mathrm{GP}}^{\prime }$ (Fig. 2A and B). $G_{\mathrm{GP}}^{\prime }$, in turn, could be adjusted over the range of 0.5 to 2.5 kPa, independently of *f_GP_*, by modulating the Gtn concentration with fixed PBA:FPBA ratio (Fig. 2B). The hydrogel was functionalized for timed release of EGCG by bonding it to phenylboronic acid. At concentrations below 5 mM, EGCG loading did not affect hydrogel mechanics (Fig. 2C and D).

Following mechanical characterization, 2 VGtn hydrogels were selected for subsequent studies. According to previous reported theoretical models [15], for compatibility with a rat heart ($G_{\mathrm{myo}}^{\prime }$= 5 kPa, $G_{\mathrm{myo}}^{\prime }$= 2 mm, *h_graft_*= 0.3 mm), a VGtn hydrogel with 15 wt% Gtn and a ratio of FPBA:PBA:DA=2:8:10 was selected for both in vitro and in vivo experiments. These hydrogels were denoted as VGtn and VGtn-E for cases without and with EGCG, respectively, and had $G_{\mathrm{GP}}^{\prime }$= 1.3 kPa and *f_GP_*= 4 Hz. For compatibility with a porcine heart ($G_{\mathrm{myo}}^{\prime }$= 5 kPa, $G_{\mathrm{myo}}^{\prime }$= 9 mm, *h_graft_*= 1 mm), a VGtn hydrogel with 20 wt% Gtn and a ratio of FPBA:PBA:DA = 6:4:10 was selected so that $G_{\mathrm{GP}}^{\prime }$= 2.5 kPa and *f_GP_*= 1.3 Hz.

The volume of VGtn-E immersed in phosphate-buffered saline (PBS) at 37 °C increased slightly (Fig. S2A), indicating that VGtn-E does not cause cardiac tamponade or mechanical compression of the heart due to hydrogel swelling. VGtn-E could be degraded enzymatically due to the proteolytic domain on Gtn chains (Fig. S2B). In vivo subcutaneous implantation experiments have shown that the hydrogel degraded slowly in vivo and maintained for more than 2 months (Fig. S2C). In PBS at 37 °C, release of EGCG by VGtn-E was fastest over the initial 7 d, and slower and more continuous over the subsequent 3 weeks (Fig. S2D). These time scales were appropriate for release of drugs over the first 7 d following MI to scavenge ROS and inhibit inflammation.

### VGtn-E is biocompatible and does not interfere with cardiac function

In vitro, primary cardiac fibroblasts (CFs), cardiomyocytes (CMs), and bone marrow mesenchymal stem cells (bMSCs) with VGtn-E extracts showed >93% viability (Fig. S3A). In vivo, VGtn-E loaded with bMSCs (denoted as VGtn-EC) injected on the epicardium of healthy rats affected neither cardiac electrophysiology nor ejection fraction, the latter measured by tracking left ventricular internal diastolic diameter (LVIDd), left ventricular internal systolic diameter (LVIDs), left ventricular ejection fraction (LVEF), and left ventricular fractional shortening (LVFS) (Fig. 2E). VGtn-EC patches were not associated with significant pathological changes to other tissues 2 months after implantation (Fig. 2F). In addition, the process of mixing and extruding VGtn-E loaded with bMSCs using a double syringe does not result in significant cell death (Fig. S3B). Scanning electron microscopy (SEM) analysis of the hydrogels shows that the pore sizes of VGtn-E hydrogels with different FPBA/PBA ratio have no significant difference, averaging around 20 μm, which is suitable for cell growth (Fig. S3C and D). These results indicate that VGtn-E hydrogel is suitable for use as a cell carrier in cardiac therapy.

### VGtn-E exhibits tough epicardial adhesion following injection

**Fig. 3.**
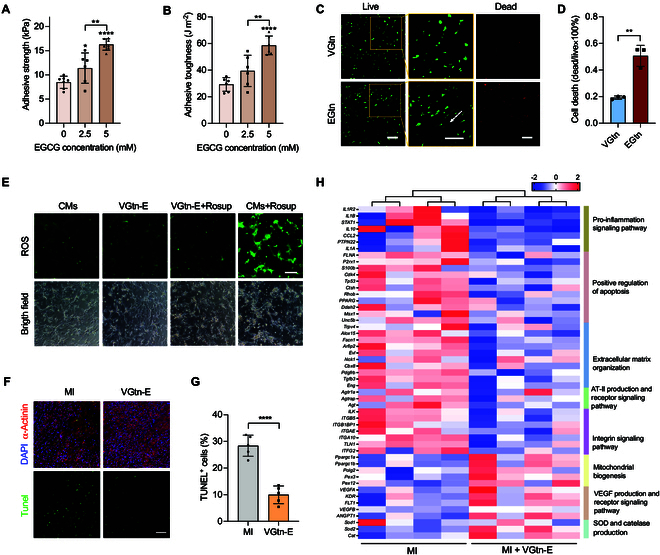
VGtn hydrogel protects cells from mechanical and ROS injury in vivo and in vitro. (A and B) Adhesive strength and toughness of VGtn-E–epicardium interface. (C) Live/dead viability staining of MSCs encapsulated in VGtn and EGtn hydrogels after 30% cyclic stretch at 4 Hz for 6 h (green, live; red, dead). The arrow in the magnified area highlights the emerging local direction of cell arrangement in EGtn. Scale bar, 100 μm. (D) Cell death ratio quantified from the image in (C). *n* = 3 independent samples for each group. (E) In vitro intracellular ROS scavenging of VGtn-E. Intracellular ROS was stained with DCFH-DA (green). Scale bar, 50 μm. (F) TUNEL staining of heart section (MI and VGtn-E) to assess CM apoptosis on day 3 after MI (TUNEL, green; α-actinin, red; DAPI, blue). Scale bar, 100 μm. (G) Quantification of TUNEL-positive cells according to fluorescence images in (F). (H) DEGs between the MI and MI treated with VGtn-E (red, up-regulated; blue, down-regulated; *n* = 4 independent samples for each group). All data are shown as means ± SD. In (A) and (B), data were compared using one-way ANOVA followed by Bonferroni’s post hoc test. In (D) and (G), data were compared using a 2-tailed Student’s *t* test. *P < 0.05, **P < 0.01, and ****P < 0.0001.

To avoid complications associated with injecting material into the myocardium or suturing the material to the surface of the myocardium [21,25], we made VGtn-E injectable and adhesive to the surface of the myocardium. Adhesion achieved via the catechol groups of DA and EGCG in VGtn-E, specifically the wet adhesivity of the diphenol group [26], yielded an adhesive strength of 16 kPa and adhesive toughness of 59 J m^−2^ on the porcine epicardium at an EGCG concentration of 5 mM (Fig. 3A and B). Injectability achieved via dynamic phenylborate crosslinks enabled the shear-thinning (Fig. S4A and B and Movie S1) and self-healing behaviors (Fig. S4C and D) to maintain structural integrity after injection. To promote cell survival during injection [27,28], we reduced shear stresses on cells by injecting the 2-component gel precursor solution via double syringes and a static mixer (Fig. 1). The precursor solution was mixed at the outlet and gelled and adhered to the epicardium within a few seconds, which can withstand flushing by running water (Fig. S4E and Movie S2).

### VGtn hydrogels exhibits stress relaxation and prestress convergence to cardiac contraction

Dynamically cross-linked hydrogels typically exhibit stress relaxation effects, which can help reduce the prestress generated during the application of the patch. We conducted stress relaxation tests on hydrogels with different ratios of Gtn-FPBA to Gtn-PBA. The results indicated that as the proportion of Gtn-PBA increased, the characteristic relaxation time of the hydrogels gradually decreased (Fig. S5A and B). To verify that VGtn-E would relax prestress while maintaining structural integrity when relaxing over normal cardiac loading cycles of ~30% strain [29], cyclic tensile tests were performed at 75% prestrain. Stress converged to a steady state after 50 cycles at 1.25 Hz (human heart rate) or 1,000 cycles at 4 Hz (rat heart rate) (Fig. S5C and D). Compared to the widely reported stiff elastic hydrogel patches with a modulus close to or greater than that of myocardial tissue, we hypothesized that VGtn with proper modulus and stress relaxation could protect cells from overstretching-related damage, based on the principle that cells are more likely to deform in stiffer extracellular matrix (ECM) [30,31]. To test this, we encapsulated bMSCs in VGtn, applied 30% uniaxial stretch at 4 Hz, and compared cell survival to that of cells in a similarly loaded, elastic Gtn hydrogel patches. This elastic control (denoted EGtn) was synthesized using methacrylated Gtn-PBA and Gtn-DA (to eliminate effects of PBA and DA, see Fig. S1) and covalently photo-crosslinked using 405-nm light in the presence of lithium phenyl(2,4,6-trimethylbenzoyl) phosphonate (LAP) to reach the shear modulus *G*^′^ = 7 kPa (Young’s modulus ~20 kPa) of a previously reported myocardial patch [32,33] (Fig. S6). Cells stretched in EGtn showed significantly lower survival than those stretched in VGtn (Fig. 3C and D), supporting the hypothesis.

### VGtn-E patches can scavenge oxygen species and reduce initial myocardial injury after acute MI

Because polyphenol groups in DA and free EGCG are dissociated dynamically by boronate esters, we expected sustained release of EGCG to scavenge ROSs. To test this, we first evaluated the total antioxidant capacity of VGtn-E. VGtn-E exhibited increased antioxidant activity with increasing EGCG concentration (Fig. S7). We soaked VGtn-E hydrogel in the medium for 24 h and cultured CMs with the lixivium. In CMs where ROSs were up-regulated by the Rosup agent, which can stimulate cells to produce intracellular ROSs, VGtn-E extract significantly inhibited intracellular ROSs (Fig. 3E), further suggesting therapeutic potential for VGtn-E.

To assess the myocardial protective effect of VGtn-E in the acute inflammatory phase after MI, we collected ischemic myocardium from rats that were given induced MI via left anterior descending artery (LAD) ligation. One group (“MI”) was not treated, and another was immediately treated with injected VGtn-E. This ischemic myocardium was harvested 3 d postoperatively, and cell apoptosis of the 2 groups was compared. At the border between the infarcted area and normal myocardial tissue (manifested by ordered CM staining and disordered cell staining), terminal deoxynucleotidyl transferase-mediated deoxyuridine triphosphate nick end labeling (TUNEL) indicated that cell apoptosis decreased in the VGtn-E group (Fig. 3F and G), indicating that VGtn-E can reduce initial myocardial injury after acute MI. To fully evaluate the protection mechanism of VGtn-E, we carried out whole-transcriptome sequencing (RNA-Seq). Kyoto Encyclopedia of Genes and Genomes (KEGG) enrichment analysis indicated VGtn-E treatment to be associated with up-regulation of fatty acid, propanoate, and carbon metabolism pathways related to myocardial function (Fig. S8A) [34,35]. VGtn-E treatment down-regulated genes involved in the promotion of apoptosis, pro-inflammatory factors, and angiotensin II, secretion of extracellular matrix, and integrin signaling, while it up-regulated genes involved in mitochondrial biogenesis and angiogenesis (Fig. 3H). In particular, gene expression of superoxide dismutase (SOD) and catalase was elevated in the VGtn-E group, suggesting that VGtn-E contributes to the scavenging of ROSs in vivo. To further demonstrate the anti-inflammatory effect of VGtn-E, we extracted rat serum 1 d after MI for enzyme-linked immunosorbent assay (ELISA) testing. The results indicated that, compared to the MI group, the serum concentrations of IL-1β, IL-10, and tumor necrosis factor-α (TNF-α) were significantly reduced in the VGtn-E group (Fig. S8B).

### VGtn-EC patches reduce LV remodeling after acute MI in a rat model

**Fig. 4.**
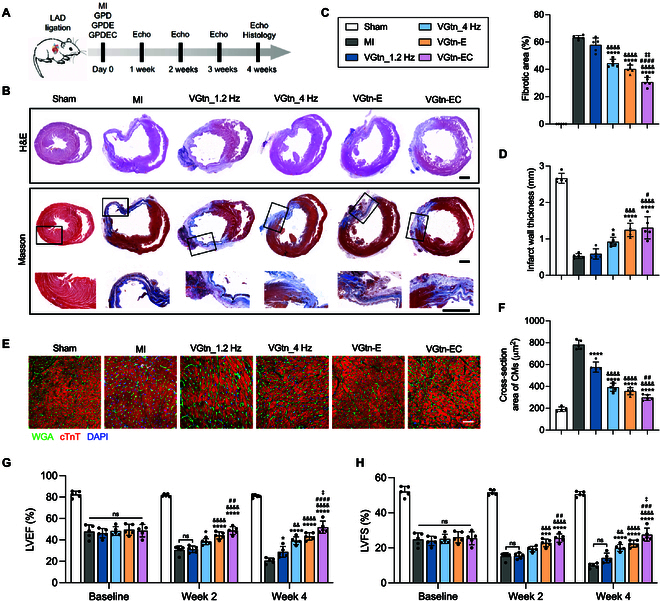
VGtn improves cardiac function and restrains LV remodeling after MI in a rat model. (A) Schematic of experimental design. (B) Representative H&E staining and Masson’s trichrome staining of heart sections for each group (Sham, MI, VGtn_1Hz VGtn_4Hz, VGtn-E, and VGtn-EC) on day 28. Scale bar, 2 mm. (C and D) Quantification of fibrotic area percentage and minimum LV wall thickness. *n* = 5 independent samples for each group. (E) Fluorescent staining of a heart section with WGA (green), cTnT (red), and DAPI (blue) to visualize CM section on day 28. Scale bar, 50 μm. (F) Quantification of the cross-sectional area of CMs in different groups according to fluorescence images in (E). *n* = 5 independent samples for each group. (G and H) Critical heart function indices (LVEF and LVFS) assessed by echocardiography. *n* = 5 independent samples for each group. All data are shown as mean ± SD. In (C), (D), and (F), data are compared using one-way ANOVA followed by Bonferroni’s post hoc test. In (G) and (H), data are compared using 2-way ANOVA followed by Bonferroni’s post hoc test. *P < 0.05, ***P < 0.001, and ****P < 0.0001 compared to the MI group; ^&&^*P* < 0.01, ^&&&^*P* < 0.001, and ^&&&&^*P* < 0.0001 compared to the VGtn_1Hz group; ^#^*P* < 0.05, ^##^*P* < 0.01, ^###^*P* < 0.001, and ^####^*P* < 0.0001 compared to the VGtn_4Hz group; ^‡^*P* < 0.05 and ^‡‡^*P* < 0.01 compared to the VGtn-E group.

To evaluate the cardioprotective effect of heart rate matched hydrogels and the mechano-biochemical synergistic therapeutic effect of VGtn patches, rats were randomly divided into 6 groups: “Sham” (thoracotomy only), “MI” (with no hydrogel treatment), “VGtn_1Hz” (*f_GP_* = 1 Hz), “VGtn_4Hz” (*f_GP_* = 4 Hz, matched with heart rate of rat), “VGtn-E”, and “VGtn-EC”. We monitored heart function by echocardiography and harvested hearts for histological evaluation after 4 weeks (Fig. 4A). Hematoxylin and eosin (H&E) and Masson’s trichrome staining revealed reduced pathological LV remodeling in hydrogel-treated groups, except for VGtn_1Hz (Fig. 4B), including reduced fibrotic area and increased LV wall thickness (Fig. 4C and D). The cross-sectional area of CMs was significantly lower in the hydrogel transplantation group, suggesting that hydrogel patch could improve myocardial hypertrophy due to pathological remodeling after myocardial necrosis (Fig. 4E and F), consistent with pathological remodeling observed after myocardial necrosis. Heart function (LVEF, LVFS) assessed by echocardiography showed further deterioration in the MI group after MI, but alleviated in the VGtn_4Hz, VGtn-E, and VGtn-EC groups (Fig. 4G and H and Fig. S9A). For the mechanically treated group, the heart rate mismatched VGtn_1Hz group showed little improvement in post-infarction sequelae and only a small reduction in LVEF loss at week 4, while the heart rate matched VGtn_4Hz group significantly improved cardiac function and reduced tissue fibrosis and myocardial hypertrophy compared to the VGtn_1Hz group. Further, VGtn-EC group with synergistic treatment achieved the best treatment outcome relative to the other groups, which may be due to the involvement of stem cells in myocardial repair through mechanisms such as paracrine signaling and exosomes [20,36]. These results highlight the potential of VGtn hydrogel in integrating mechanotherapy and biochemical therapy for myocardial protection.

To assess revascularization, which is essential for recovery from myocardial ischemic injury, we analyzed angiogenesis by immunofluorescence staining for α-smooth muscle actin (α-SMA), CD31, and CD34 at day 28 after MI (Fig. S9B). Compared with the MI group, angiogenesis was unchanged in the VGtn_1Hz and VGtn_4Hz group, increased in the VGtn-E group, and further increased in the VGtn-EC group (Fig. S9C to E). These results were consistent with earlier observations that biochemical factors dominate over mechanical factors in promoting angiogenesis [15,21], and underscored the importance of multifactorial, synergistic treatment.

### VGtn patches improve function in advanced fibrotic cardiomyopathy by coordinating LV mechanical contraction

**Fig. 5.**
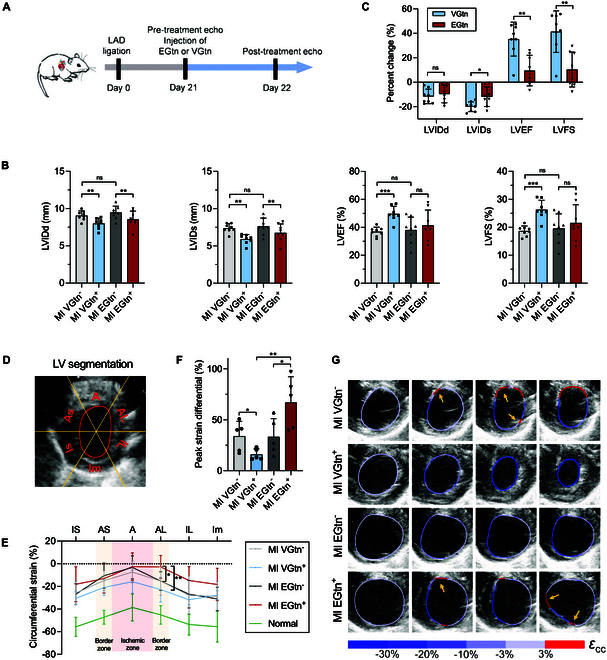
VGtn improves ventricular ejection of the fibrotic heart by coordinating LV systole. (A) Schematic of experimental design. (B) Critical heart function indices (LVIDd, LVIDs, LVEF, and LVFS) of fibrotic hearts assessed by echocardiography before and after VGtn or EGtn implantation. *n* = 8 independent samples for each hydrogel group. Data are compared using paired *t* tests within each hydrogel group and a 2-tailed Student’s *t* test between different hydrogel groups. (C) Heart function changes after hydrogel treatment. (D) Ultrasonography and segmentation diagram of the LV. (E) Spatial variations across LV segments. *n* = 5 independent samples for each hydrogel group and *n* = 5 for the normal control group. (F) Maximum peak strain differential *Δε_cc_* over the course of a cardiac cycle. *n* = 5 independent samples for each group; *Δε_cc_* serves as a proxy for the magnitude of the strain gradient. Note that MI VGtn^−^ indicates responses of the heart at 21 d after infarct, just before injection of VGtn, and MI VGtn^+^ indicates responses 1 d later; similarly, MI EGtn^−^ and EGtn^+^ indicate, respectively, responses 21 d after infarct (just before injection of EGtn) and at 22 d. (G) Spatial distribution of *ε*_cc_ in the LV over 4 consecutive images during a systolic cycle. Yellow arrows denote the transition between stretching and contraction, where the strain gradient is largest. All data are shown as mean ± SD and compared using 2-tailed Student’s *t* tests unless otherwise stated. *P < 0.05, **P < 0.01, and ***P < 0.001.

Progressive fibrotic cardiomyopathy can, in its later stages, lead to ventricular wall thinning and poor prognosis, including risks of ventricular aneurysm and rupture. The LV wall can be strengthened in advanced fibrotic cardiomyopathy using stiff and elastic patches, but at the expense of restricting cardiac contraction, relaxation, and ejection [12,13,37]. To explore the mechanical benefits of VGtn in advanced fibrotic hearts while excluding biochemical factors, we performed ultrasound assessments on rats 21 d after MI. VGtn and EGtn were then injected into the untreated myocardial fibrotic regions of the rats, identified by whitened and thinned tissue, followed by a second ultrasound assessment on the same rat 1 d after treatment (Fig. 5A). Echocardiography showed that VGtn and EGtn injection both reduced ventricular dilatations, as reflected by reduced LVIDd and LVIDs (Fig. 5B). However, as expected because of its tuned viscoelasticity, VGtn substantially outperformed EGtn in improving systolic function, as reflected by improved LVIDs (Fig. 5C), and significantly improved ventricular ejection (LVEF and LVFS) (Fig. 5B and C).

To further assess the effect of epicardial patches on myocardial contraction, we calculated the circumferential contractile strain (*ε*_cc_) in the mid-segment of the LV short axis (Fig. 5D) from echocardiography images. *ε*_cc_, averaged over the entire LV, reduced in magnitude relative to healthy controls in mice with MI (Fig. 5E). VGtn injection was associated with a slight increase in *ε*_cc_ magnitude in the infarct zone without any change in the healthy tissue. In contrast, *ε*_cc_ in the infarct zone did not improve after EGtn implantation, and the mean contractile strain in neighboring zone decreased. This suggested that the stiff elastic patches may restrain myocardial contraction in the covered area.

The border zone between the noncontractile infarct zone (fibrotic tissue) and the adjacent contractile myocardium experiences relatively high gradients in strain [38] and is associated with increased risk of wall rupture. To quantify the strain gradient at the infarct boundary, we subdivided the heart wall into 24 equal parts based on the classic 6-segment segmentation and calculated the strain differential (Δ*ε*_cc_) as the magnitude of the difference between *ε*_cc_ in adjacent partitions. Δ*ε*_cc_ was highest at the infarct border and was reduced by treatment with VGtn but increased by treatment with EGtn (Fig. 5F). EGtn led to tensile strain slightly further away from the infarct border (Fig. 5G), consistent with stress concentrations associated with material mismatches [39]. Results suggested that VGtn can improve coordination of LV contraction, reduce strain gradients at the infarct border, and thereby improve ventricular ejection in hearts with advanced fibrosis.

### VGtn hydrogel patches improve heart function in pigs after MI

**Fig. 6.**
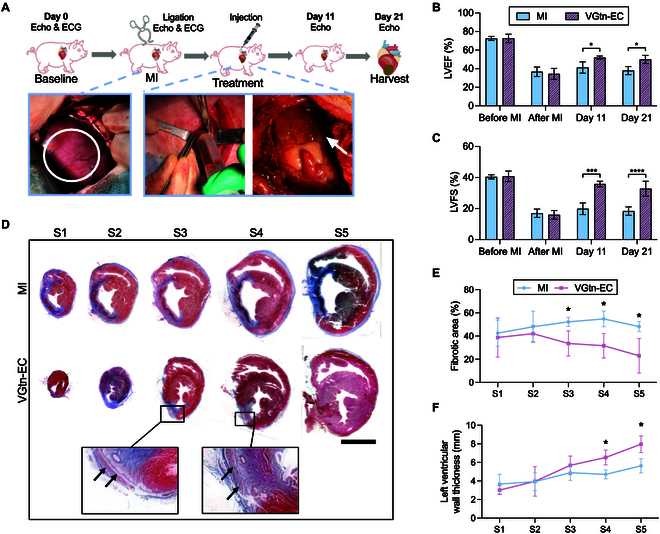
VGtn-EC improves cardiac function after MI in a porcine model. (A) Schematic of experimental and surgical design. Left picture: ischemic myocardium after LAD ligation (white circle); center picture: injection of VGtn-EC; right picture: VGtn-EC adhered to the epicardium at animal sacrifice on day 21 (white arrow). (B and C) LVEF and LVFS of pigs assessed by echocardiography. *n* = 3 independent samples for each group. (D) Representative Masson’s trichrome staining of heart sections on day 21. Hearts were cut into 5 slices (S1 to S5) from the apex to the point of ligation. Black arrows denote injected VGtn-EC. Scale bar, 2 cm. (E and F) Quantification of infarction area percentage and average LV wall thickness on day 21. *n* = 3 independent samples for each group. All data are shown as mean ± SD and compared using 2-tailed Student’s *t* tests. *P < 0.05, ***P < 0.001, and ****P < 0.0001.

To evaluate the potential for clinical translation, we tuned these patches to a porcine heart (VGtn-EC with $G_{\mathrm{GP}}^{\prime }$ of 2.5 kPa and *f_GP_* of 1.3 Hz) and assessed their efficacy in a porcine model of MI. LAD ligation was performed via thoracotomy, with induction of MI confirmed by electrocardiogram (ECG) (elevation of the ST segment), echocardiography, and increased serum levels of cardiac troponin I (cTnI) (Figs. S10 and S11A). A double syringe with a static mixer was used to inject the hydrogel evenly on the epicardium (Fig. 6A), and therapeutic effects were compared to those in a sham treatment control group.

Echocardiography showed that VGtn-EC treatment significantly improved cardiac ejection (LVEF) and systolic function (LVFS) (Fig. 6B and C and Fig. S11A). Examination of hearts harvested on day 21 showed additional signs of augmented healing. Five transverse slices (S1 to S5) of the heart from the apex to the ligation site (10 mm per slice) were studied. Both Masson’s trichrome staining and direct view photographs showed that VGtn-EC was localized between the pericardium and the ventricular wall (Fig. 6D and Fig. S11B). VGtn-EC reduced LV remodeling and fibrotic area (Fig. 6E and F). α-SMA immunostaining demonstrated enhanced angiogenesis after VGtn-EC treatment (Fig. S11C and D).

To evaluate the biological safety of VGtn-EC, we collected blood samples at 2 time points (before MI and day 21) and assessed the hepatotoxicity and nephrotoxicity of VGtn-EC. The serum concentrations of alanine aminotransferase (ALT), direct bilirubin (DBIL), blood creatinine (CREA), and urea nitrogen (UREA) did not change significantly (Fig. S12). Overall, these results supported the effectiveness and safety of VGtn-EC in porcine MI models.

## Discussion

Myocardial patches are desirable for delivering therapeutic factors that attenuate fibrosis and for reinforcing the infarcted ventricular wall after MI. Although hydrogel-based treatments are mature for the delivery of therapeutic factors, all patches have a potentially negative biomechanical influence that has only recently been identified [13,15,21,40]. Our viscoelastic hydrogels with independently tunable $G_{\mathrm{GP}}^{\prime }$ and *f_GP_* showed potential for overcoming these negative effects in a way that could be tuned to specific species, and potentially to specific patients. Our results validate a previous theoretical prediction [15] that a heart rate matched viscoelastic hydrogel would have multiple mechanical benefit on MI treatment compared with heart rate mismatched hydrogel (Fig. 4). Here, such a hydrogel coordinated myocardial contraction, reduced the strain gradient at the infarct border, and supported polyphenol drugs and stem cell therapy to reduce myocardial apoptosis, inhibit inflammation, and promote vascularization, thereby reducing the occurrence and expansion of fibrosis and improving ventricular ejection of advanced cardiac fibrosis.

Hydrogels based on boronate esters have been extensively studied in biomedical applications [41]. In this work, we focus on its intriguing characteristic time scales, which can be intricately matched to the time scale of physiological activities, allowing for precise tuning through the selection of different types of phenylboronic acids. On the other hand, the introduction of polyphenol groups not only enhances the adhesion of hydrogels to tissues but also provides antioxidant and anti-inflammatory effects. Numerous studies have used DA-modified biomacromolecules or DA derivatives (e.g., polydopamine nanoparticles) for myocardial repair [42,43], demonstrating the biosafety of this approach. Therefore, selecting phenylboronic acid and polyphenols as the crosslinking units of the VGtn hydrogel is a rational choice that balances both mechanical and biochemical therapies.

In addition to inhibiting LV dilatation and LV rupture, epicardial patches may have additional therapeutic implications assorted with coordinating strains at infarct borders. Despite the lack of direct evidence in vivo, in vitro culture models suggest that different mechanical factors, including high strain gradients and passive stretching in the infarct boundary area, may promote activation of fibroblasts and spreading of fibrosis beyond the infarction zone [44–47]. Myocardial strain assessment in hearts with advanced fibrosis (Fig. 5G) suggests that VGtn may attenuate the expansion of fibrosis by dispersing stress concentrations in the border zone and reducing passive stretch of healthy tissue, potentially reducing the risk of fibrotic transformation in the surrounding myocardium.

VGtn showed benefit in animal models of advanced fibrotic cardiomyopathy, including strengthening ventricular wall, which may rupture and cause sudden death [48,49]. Previously reported elastic patches or meshes to reinforce infarcted myocardium have reduced the incidence of rupture, but this came at the cost of downstream complications [50–52]. Our tuned viscoelastic patches attenuated complications of advanced fibrosis and improved ventricular ejection. Pericardial injection of VGtn hydrogel patches may offer a treatment pathway that has benefits over the current standard of care, surgical ventricular reconstruction [53,54], including reduced invasiveness and faster patient responses.

Loading hydrogels with bMSCs in addition to specific drugs showed multiple synergistic benefits. Although cell therapies immediately following a heart attack are not yet feasible clinically, these results support the use of a combined mechano-biochemical approach. This viscoelastic platform could be further combined with other biophysical cues (conductivity [55]) or emerging therapeutic factors (e.g., exosomes [56], RNA [57], and engineered induced pluripotent stem cells (iPSCs) [58]) to complement the treatment of ischemic heart disease. Together, these highlight clinical potential for VGtn hydrogel patches to improve cardiac function after MI. Although these results are still preclinical, they do suggest promise for future efforts to move such technologies toward the clinic.

## Materials and Methods

### Synthesis of hydrogels

Gtn-PBA and Gtn-FPBA precursors were synthesized through ethyl-dimethyl-aminopropyl carbodiimide (EDC; Aladdin) and *N*-hydroxy-succinimide (NHS; Aladdin) coupling chemistry. Briefly, type A Gtn (1 g, Sigma-Aldrich) was dissolved in 40 ml of D-H solvent (dimethyl sulfoxide/H_2_O = 1:1) at 40 °C. PBA or FPBA (4 mM) was dissolved in 20 ml of D-H solvent in a centrifuge tube. EDC and NHS were added to the tube until the final molar ratio of PBA/EDC/NHS was set to 1:1.2:1.4. The mixture was allowed to react for 30 min at room temperature to activate the carboxyl groups and then added to the Gtn solution. The solution pH was maintained from 4.5 to 5.5 for 24 h using 1 M NaOH. After the reaction, the solution was purified by dialysis against 1% Na_2_CO_3_ solution for 1 d and ultrapure water for 3 d and then lyophilized. Gtn-DA precursor was synthesized through EDC/NHS reaction using DA hydrochloride (Aladdin) according to previously reported protocols [59]. For elastic Gtn hydrogel (EGtn), Gtn was replaced by an equivalent amount of gelatin methacryloyl (GelMA) [degree of substitution (DS): 25%], which was synthesized using methacrylic anhydride (MA; Aladdin) as previously reported [60]. To confirm successful grafting, the precursors were analyzed by proton nuclear magnetic resonance (^1^H NMR) using a 400-MHz JEOL nuclear magnetic resonance spectrometer (Fig. S1).

The successful modification of each component is indicated by the characteristic peaks of ^1^H NMR spectra. The peak area of lysine methylene protons appearing between ≈2.9 and 3.1 parts per million (ppm) was used for the calculation of the DS. The peaks corresponding to different groups include PBA (phenylboronic group, between ≈7.36 and 7.79 ppm, -C_6_H_4_, DS ≈ 67%), FPBA (fluoro-phenylboronic group, between ≈7.51 and 7.91 ppm, -C_6_H_3_F, DS ≈ 75%), DA (catechol groups, between ≈6.89 and 7.21 ppm, -C_6_H_4_O_2_), and methacryloyl group (between ≈5.4 and 5.8 ppm, DS ≈ 34%). The substitution of DA groups was determined by Arnow’s method as previously reported [61], with a concentration of 61.82 μmol/g.

To form VGtn hydrogels, the Gtn-PBA, Gtn-FPBA, and Gtn-DA precursors were dissolved in phosphate-buffered saline (PBS), then mixed according to the given ratio in a tube, and vortexed. For VGtn-E hydrogels, Gtn-DA was dissolved in PBS containing 5 mM EGCG. To form EGtn hydrogels, 0.2% LAP (Aladdin) was introduced to precursors and exposed to blue light irradiation (405 nm) at an intensity of 100 mW/cm^2^ for 20 s. For cell culture and animal experiments, all hydrogel precursors were filtrated through a 0.22-μm membrane filter (Millex-GP; Millipore).

### Mechanical characterization

For rheology tests, an MCR 302 rheometer (Anton Paar) with 15-mm stainless steel parallel plates was used. Samples were prepared by mixing precursors on the plate at a thickness of 0.8 mm and waiting 10 min to ensure full crosslinking. Then, a frequency sweep test was performed on the hydrogels at a strain of 1% and frequency from 0.1 to 10 Hz. Strain sweeps were performed in advance to confirm that the strain was within the linear elastic regime. $G_{\mathrm{GP}}^{\prime }$ was calculated by fitting the modulus curve using a custom Matlab script (The Mathworks, Natick, MA). For the stress relaxation tests, the hydrogel was subjected to a 10% strain and allowed to relax for over 50 s, and stress changes were continuously recorded. The stress relaxation curve was fitted using a second-order Maxwell model.

To quantify self-healing of the hydrogels, 4 cycles of a time sweep at 1 Hz were performed (200% strain for 1 min and 1% strain for 5 min). To test the mechanical adaptivity of hydrogels, we carried out cyclic stretching tests using a universal tensile testing machine (Bose). Hydrogels were prepared in a strip mold with a length of 10 mm, a width of 15 mm, and a thickness of 2 mm. The strips were fixed on a stretching clamp at a spacing of about 6 mm. The strips were initially stretched to 10 mm (about 75% per strain, 4 mm/s) to mimic the prestretch. Then, we carried out a cyclic stretching between 8 mm (set as the 0 strain) and 10 mm (25% strain) for 1,000 cycles at a frequency of 1.25 Hz or 4 Hz.

### Characterization of adhesive properties of the hydrogels

We first trimmed fresh myocardial tissue with porcine epicardium into discs with a diameter of 20 mm and fixed them to both the upper and lower flat indenters of a universal tensile-testing machine. Then, we placed the hydrogels on the epicardium on the lower plate and lowered the upper plate to form a sandwich structure. After stabilizing for 1 min, we separate the upper and lower plates at a speed of 1 mm/s and recorded the force–displacement curve. Adhesive strength was defined as the maximum stress reached prior to failure, and adhesive toughness was calculated as integral of the stress–displacement curve. In all cases, failure occurred by debonding rather than by fracture of the adhesive itself.

### Swelling, degradation, and injectability

To characterize swelling of the hydrogels, we measured wet weights of the hydrogels after preparation and after incubating in PBS at 37 °C for 7, 30, and 60 d. To characterize enzyme degradation of the hydrogels, hydrogels were soaked in PBS with type II collagenase [MP Biomedicals, 100 U per patch (100 μl of hydrogel)] at 37 °C and recorded the wet weights. To characterize injectability, the formed hydrogels were injected into the water through a 25-gauge needle.

### Characterization of antioxidant function

2,2’-Azino-bis(3-ethylbenzthiazoline-6-sulfonic acid (ABTS) was used to evaluate the antioxidant function of the hydrogels with a total antioxidant capacity assay kit (Beyotime Biotechnology, Shanghai, China) according to the manufacturer’s instructions. Briefly, 10 μl of hydrogels containing different concentrations of EGCG was homogenized in 200 μl of PBS, and then 10 μl of homogenate was added to the working solution of the kit. A microplate reader was used to detect the absorbance at 414 nm. This was compared to the Trolox standard curve to calculate the Trolox equivalent antioxidant capacity (TEAC). Quantification of intracellular ROS scavenging was carried out using a ROS assay kit (Beyotime Biotechnology) according to the manufacturer’s instructions. Primary cardiac myocytes (1 × 10^6^ cells per well) were seeded in a 6-well plate fed with serum-free medium or medium soaked with Gtn-E hydrogels (5 mM EGCG, 50 μl of gel per 1 ml of medium) for 1 h. Subsequently, cells were treated with 2′,7′-dichlorodihydrofluorescein diacetate (DCFH-DA, 1 μl/ml, 30 min) and imaged with a fluorescence microscope at 488 nm. For the positive control group, cells were treated with Rosup reagent (1 μg/ml, 30 min) to raise the intracellular ROS levels.

### Characterization of in vitro EGCG release

EGCG release was conducted in 1× PBS at pH 7.4, and 1 ml of VGtn-E hydrogel (with 5 mM EGCG) was soaked in 50 ml of PBS at 37 °C. Then, 1 ml of supernatant was taken at different time points for testing (0.5, 1, 2, 3, 5, 7, 10, 15, 20, and 30 d) and supplemented with an equal volume of fresh PBS. EGCG solubility in solution was measured by an ultraviolet–visible spectrophotometer (Lambda950).

### Cell culture and in vitro experiments

Primary CFs were isolated from neonatal Sprague–Dawley rats as in our previous work [62]. Rat bMSCs and pig bMSCs were isolated by Ficoll density gradient centrifugation as previously described [63]. Briefly, fresh bone marrow was harvested from 4-week-old Sprague–Dawley rats and 3-month-old pigs. CFs were cultured in Dulbecco’s modified Eagle’s medium (DMEM)/Ham’s F-12 (1:1) (DF-12), while rat bMSCs and pig bMSCs were cultured in DMEM containing 1 g/l glucose (DMEM-LG). All culture medium was added with 10% fetal bovine serum (Gibco/Thermo Fisher Scientific) and 1% penicillin-streptomycin (Gibco/Thermo Fisher Scientific) at 37 °C in 5% CO_2_. To evaluate the cytocompatibility of the hydrogels, 1 ml of VGtn-E hydrogels was soaked in 10-ml culture medium for 24 h, and CFs, CMs, and bMSCs were then cultured with hydrogel extracts. Cell viability was evaluated using the methylthiazolyldiphenyl-tetrazolium bromide (MTT assay, Beyotime Biotechnology, China) according to the manufacturer’s instructions. For cell encapsulation, we first uniformly mixed the cells with the precursor solutions of Gtn-DA and Gtn-PBA/FPBA and then loaded the 2 precursor solutions into separate syringes of a double-syringe system. For in vitro stretching experiments, VGtn- and EGtn-encapsulated bMSCs were adhered to a polydimethylsiloxane (PDMS) film, which was stretched by a customized uniaxial stretching device [64]. After encapsulating Gtn hydrogels, cells received 30% strain at a frequency of 4 Hz, using a custom-made stretching device. After loading for 6 h, cells were immediately stained using Live/Dead Viability Kit (Invitrogen).

### Rat surgeries and injection of hydrogels

All procedures were approved by the Institutional Animal Care and Use Committee of the Air Force Medical University of the People’s Liberation Army (No. 2021001). Male Sprague–Dawley rats (Laboratory Animal Center of Xi’an Jiaotong University, China) (weight, 250 to 280 g) were given induced acute MI through LAD artery ligation. Rats were housed under constant temperature conditions (22 ± 2 °C) with a 12-h light/dark cycle and provided with standard laboratory food and ad libitum access to water. For MI model, rats were anesthetized with 1.5% sodium pentobarbital solution and mechanically ventilated through tracheal intubation. Each rat was placed on a heating pad in a supine position, where the chest fur was shaved and disinfected with povidone–iodine solution. A left thoracotomy was performed in the fourth intercostal space. After thoracotomy and removal of pericardium, the LAD coronary artery was permanently ligated with 6-0 sutures to induce MI. For the Sham group, thorax was closed after the removal of the pericardium. To prevent infections, penicillin (20,000 U/kg) was administered via intramuscular injection for 3 d postoperatively. One day after MI, echocardiography was carried out to confirm successful modeling. Then, MI rats were randomized into 5 groups: MI, VGtn_1Hz, VGtn_4Hz, VGtn-E, and VGtn-EC. For the cell loading group VGtn-EC, a bMSC suspension was mixed with the hydrogel precursors (1 × 10^6^ cells per patch) and used immediately after gelation. Hydrogel (80 μl) was injected and adhered to the infarcted area. To assess the effect of VGtn and EGtn on fibrotic hearts, rats were first given induced MI injury on day 0, and then ventricular function was checked via echocardiography at day 21. After that, a second operation was performed to inject either VGtn or EGtn, and echocardiography was repeated 1 d later. For ELISA test, serum samples of rats were collected 1 d after MI. The serum concentration of IL-1β, IL-10, and TNF-α was quantified in triplicate using ELISA kits (Proteintech, China) following the manufacturer’s instructions.

### Echocardiography

LV contractile function was examined by echocardiography on a Vevo 2200 imaging system (VisualSonics). We collected data at 3 time points (1 d after MI as baseline, week 2, and week 4). Briefly, we used 2% isoflurane inhalation to anesthetize rats and captured conventional 2-dimensional images and M-mode images of the short axis. LVEF and LVFS were determined and calculated by tracking the endocardial boundary during the contraction cycle.

### Histological assessment

Rats were euthanized, and the harvested hearts were fixed in a 4% (w/v) formaldehyde solution, paraffin embedded, and sectioned. H&E staining and Masson’s trichrome staining of sections were conducted using standard methods. The infract wall thickness was defined as the minimum value LV wall thickness. The infarction percentage was defined as (fibrosis area wall circumference/total LV wall circumference) × 100%.

### Immunofluorescence staining

Fresh hearts were embedded into optimal cutting temperature (O.C.T.; Sakura Finetek) compound, sectioned at −20 °C, and fixed with acetone. After permeabilization with 0.5% Triton X-100 (Sigma-Aldrich) for 10 min and blocking with normal goat serum for 30 min, slides were incubated with different primary antibodies at 4 °C overnight. The excess primary antibody was washed 3 times with PBS and incubated with an appropriate secondary antibody for 1 h and 4′,6-diamidino-2-phenylindole (DAPI) for 5 min at room temperature. Finally, all slides were covered with antifade mounting medium and coverslips to reduce fluorescence attenuation. Besides, some slides were counterstained with Alexa Fluor 488-conjugated wheat germ agglutinin (WGA; Thermo Fisher Scientific) or TUNEL (One Step TUNEL Apoptosis Assay Kit, Beyotime Biotechnology) according to the manufacturer’s instructions.

### RNA-Seq experiments

Total RNA was extracted from the infarct tissue in the MI and VGtn-E groups 3 d after MI using TRIzol according to the manufacturer’s protocol. The RNA quantity was evaluated with an Agilent 2100 Bioanalyzer system (Agilent Technologies) and then purified using poly-T oligolinked magnetic beads. After purification, mRNA was fragmented into small fragments using fragmentation buffer. The first-strand complementary DNA (cDNA) was synthesized using random hexamers, and the second-strand cDNA was then synthesized using deoxynucleotide triphosphates, ribonuclease H, and DNA polymerases. After repair by adding a single “A” base to the 3′ end and ligation of the adaptors, cDNAs were purified by agarose gel electrophoresis and enriched with polymerase chain reaction to obtain the final cDNA libraries, and then quantified using the Agilent 2100 system. The cDNA libraries were pooled at an equal ratio and used for 150-base pair paired-end sequencing in a single lane on HiSeq PE Cluster Kit v4-cBot-HS (Illumina) according to the manufacturer’s instructions, and the fragments per kilobase per million (FPKM) transcriptome data of 10 MI and 10 VGtn-E samples were obtained. The “edgeR” package calculation in R 3.5.2 software was used to identify the differentially expressed genes (DEGs) of the 2 groups according to the cutoff value of |log_2_FC| > 1 and *P* < 0.05. Gene ontology (GO) terms including biological processes (BPs), cellular components (CCs), and molecular functions (MFs) were analyzed by R “clusterProfiler” package to perform biological functions of DEGs between MI and VGtn-E groups. To investigate the signaling pathways enriched by differentially expressed genes (DEGs), KEGG enrichment analysis was conducted by the “clusterProfiler” package of R software with a statistical threshold of *P* < 0.05.

### Microscopy and image analysis

Unless specifically mentioned, bright-field images were taken with an Olympus IX2-UCB microscope and fluorescence images were taken with an Olympus FV3000 confocal laser-scanning microscope. The cross-sectional area of CMs was measured by WGA and cTnT costained images. CM contours were determined by WGA, and the thresholded images were inverted to enable automated counting of the cell area. TUNEL-stained images were used to evaluate cell apoptosis according to the following formula: (number of TUNEL^+^ cell nuclei/number of total cell nuclei) × 100%. All image analysis was performed by ImageJ (National Institutes of Health) unless specifically mentioned. To assess LV circumferential contractile strain, we obtained dynamic images of the LV short axis and then used a customized recognition program combined with manual correction to obtain the contour of the ventricle wall. Circumferential contractile strain (*ε*_cc_) was defined as the rate of systolic myocardial length relative to diastolic myocardial length within the same segment: (*l_systolic_* − *l_diastolic_*)/*l_diastolic_* × 100%.

### Porcine experiments

All procedures were approved by the Institutional Animal Care and Use Committee of the Air Force Medical University of the People’s Liberation Army. Pigs weighing 30 to 40 kg were injected intravenously with propofol to induce anesthesia. Anesthesia was maintained by supplying isoflurane (0% to 5% in oxygen) during the surgery. After thoracotomy, the pericardium was opened to expose the heart, and the LAD coronary artery was permanently ligated with 4-0 Prolene sutures to induce MI. The successful induction of MI was confirmed by ECG and the whitening of tissues downstream of the LAD. For the treatment group, VGtn-EC was evenly injected into the pericardial cavity and the pericardium and chest were then closed. ECG, arterial oxygen saturation, blood pressure, and heart rate were monitored throughout the procedure. Blood samples were collected before surgery, 1 d after surgery, and 21 d after surgery to confirm cTnI levels and assess liver and kidney function. Echocardiography (Mindray, China) was carried before and right after surgery, and monitored at 11 and 21 d. On the 21st day, all pigs were euthanized, and the hearts were harvested.

### Statistics

Statistical analyses were performed using GraphPad Prism 8.0. Multiple comparisons among the 3 or more groups were performed using one-way analysis of variance (ANOVA) with Bonferroni post hoc testing, and differences between 2 groups were analyzed by a 2-tailed Student’s *t* test. *P* < 0.05 was taken as the threshold for significant differences. All data are shown as mean ± SD.

## Data Availability

All relevant data supporting the key findings of this study are available within the article and its Supplementary Materials files or from the corresponding author upon reasonable request.
