# Supplementary material for: A Heart Rate Matched Patch for Mechano-Chemical Treatment of Myocardial Infarction: Optimal Design and Transspecies Application
Source: Research (Wash D C). 2024 Nov 22;7:0517. doi: 10.34133/research.0517 (PMC11582187; doi:10.34133/research.0517)
Supplement: Supplementary 1 — Figs. S1 to S12 [file research.0517.f1.zip › sopporting infromation_Revise.docx]

**Supplementary figures**


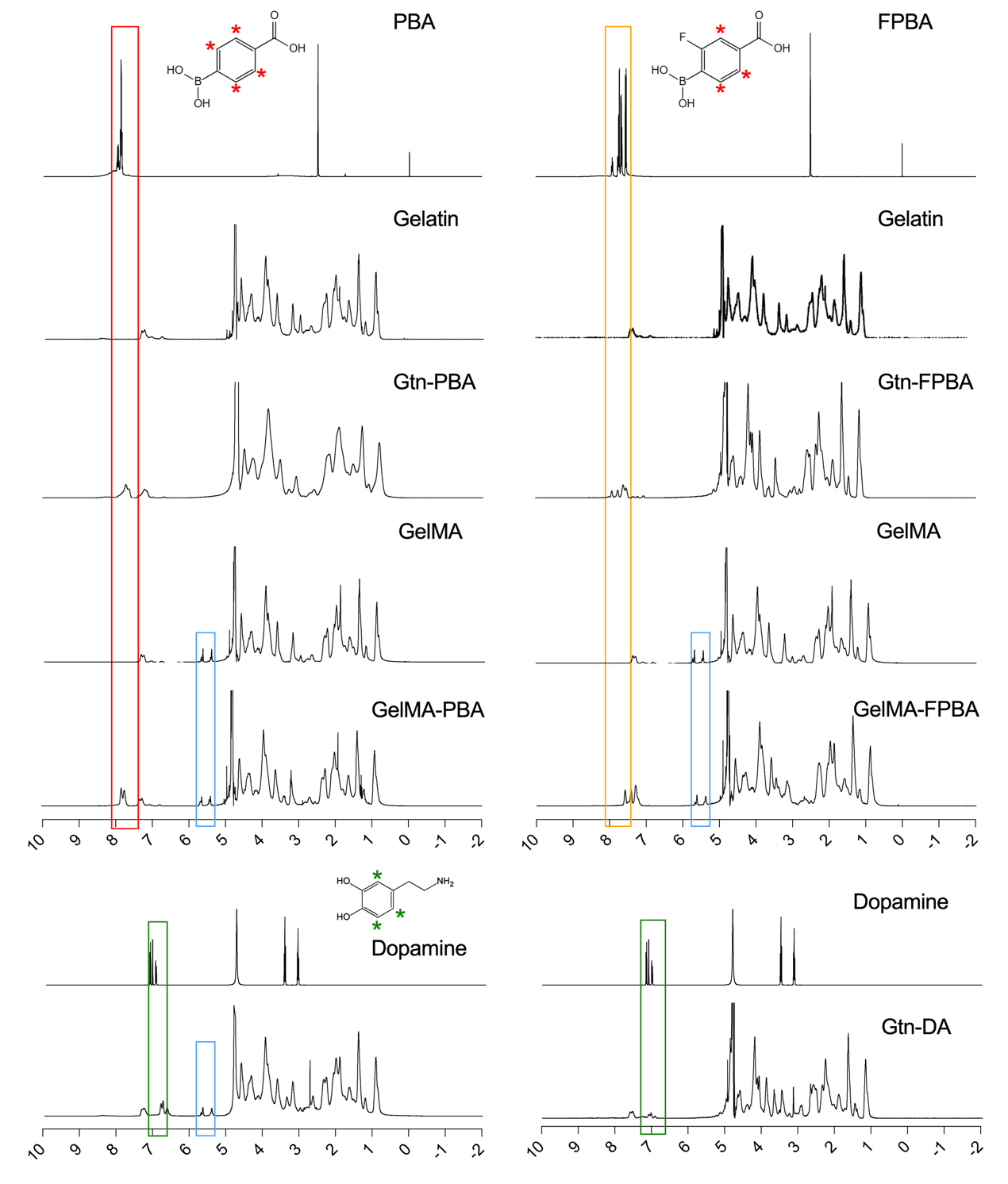


**Supplementary Figure S1 | ^1^H NMR spectra of Gtn-PBA, Gtn-FPBA, Gtn-DA, GelMA, GelMA-PBA, GelMA-FPBA, GelMA-DA.**


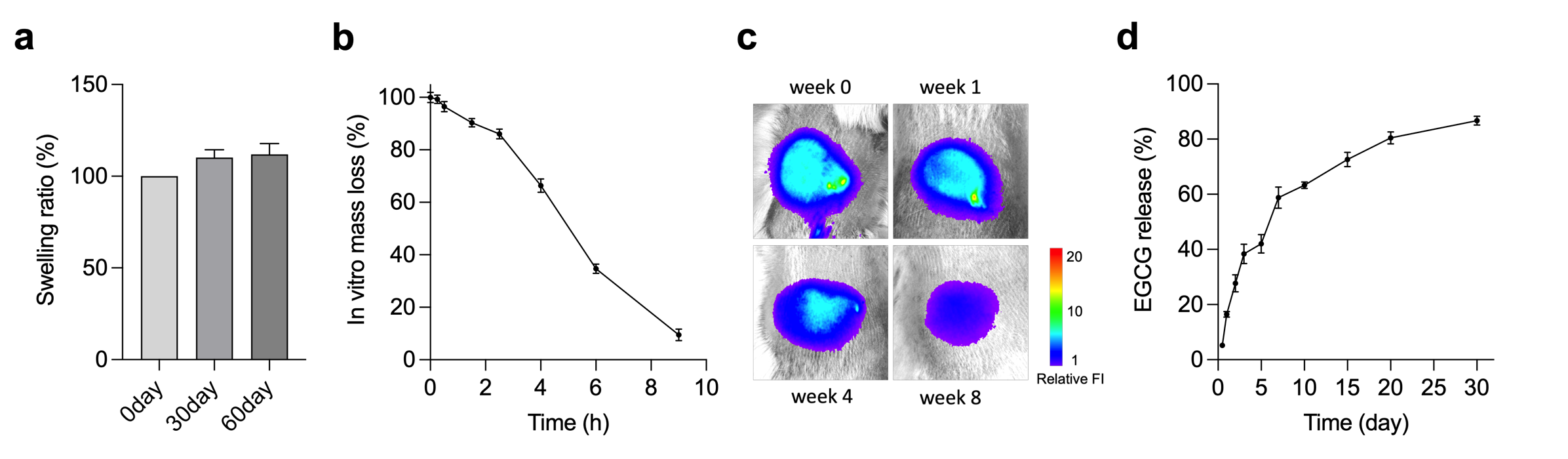


**Supplementary Figure S2 | Swelling, degradation and EGCG release of VGtn-E.**

**a,** Swelling ratio of VGtn-E in PBS for 60 days. *n*=5 independent samples for each group. Data are shown as mean ± s.d.. **b,** Type I collagenase (100U per graft) induced in vitro degradation of VGtn-E graft. **c,** In vivo imaging of FITC-labelled VGtn-E hydrogel. **d,** EGCG release curve in PBS at 37°C.


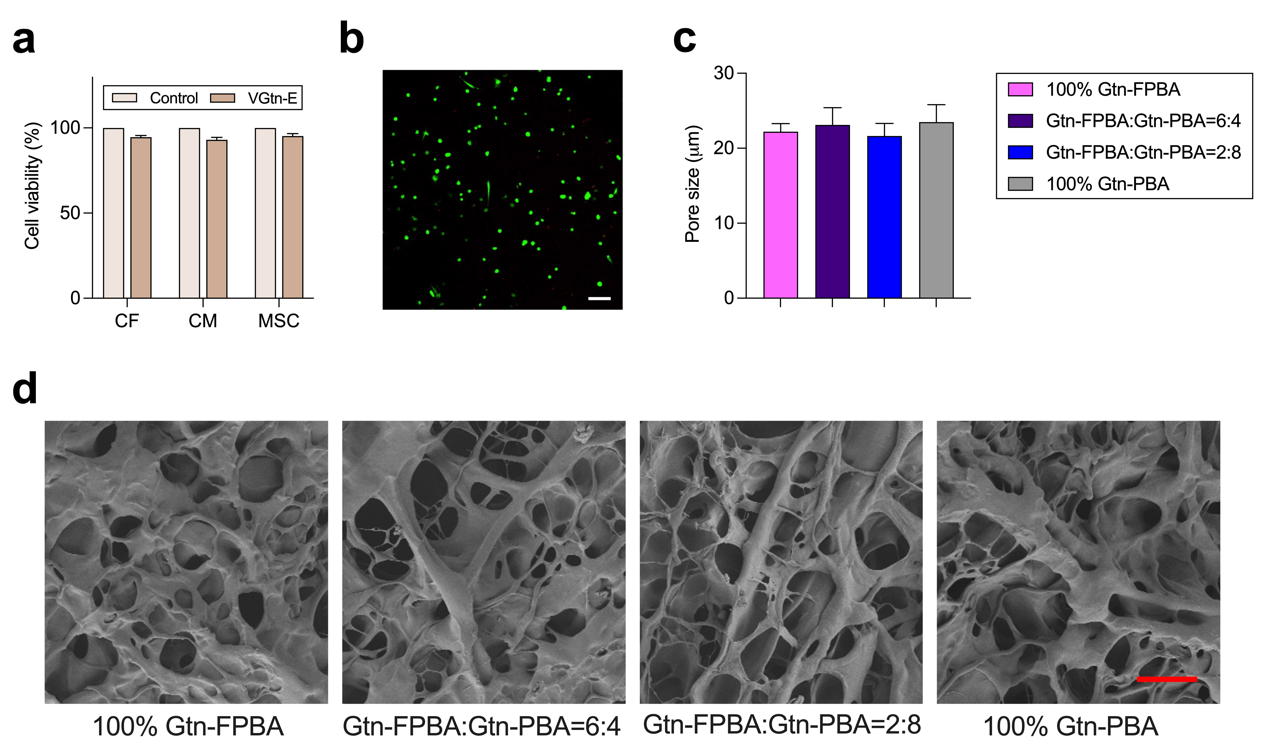


**Supplementary Figure S3 | VGtn-E had high cell compatibility.**

**a,** Cell viability quantified from MTT assay of CFs, CMs and MSCs cultured with VGtn-E extracts, *n*=3 independent samples for each group. **b,** Live/dead viability staining of MSCs after injection with a double syringe (green, live; red, dead). Scale bar, 100 μm. **c,** Pore size quantized by SEM images of d. *n*=3 independent samples for each group and compared by one-way ANOVA followed by Bonferroni’s post hoc test. No significant difference was found between the different groups. **d,** SEM images of VGtn-E hydrogels with different FPBA/PBA ratio (gelatin concentration: 15% w/v). Scale bar, 50 μm. All Data are shown as mean ± s.d..


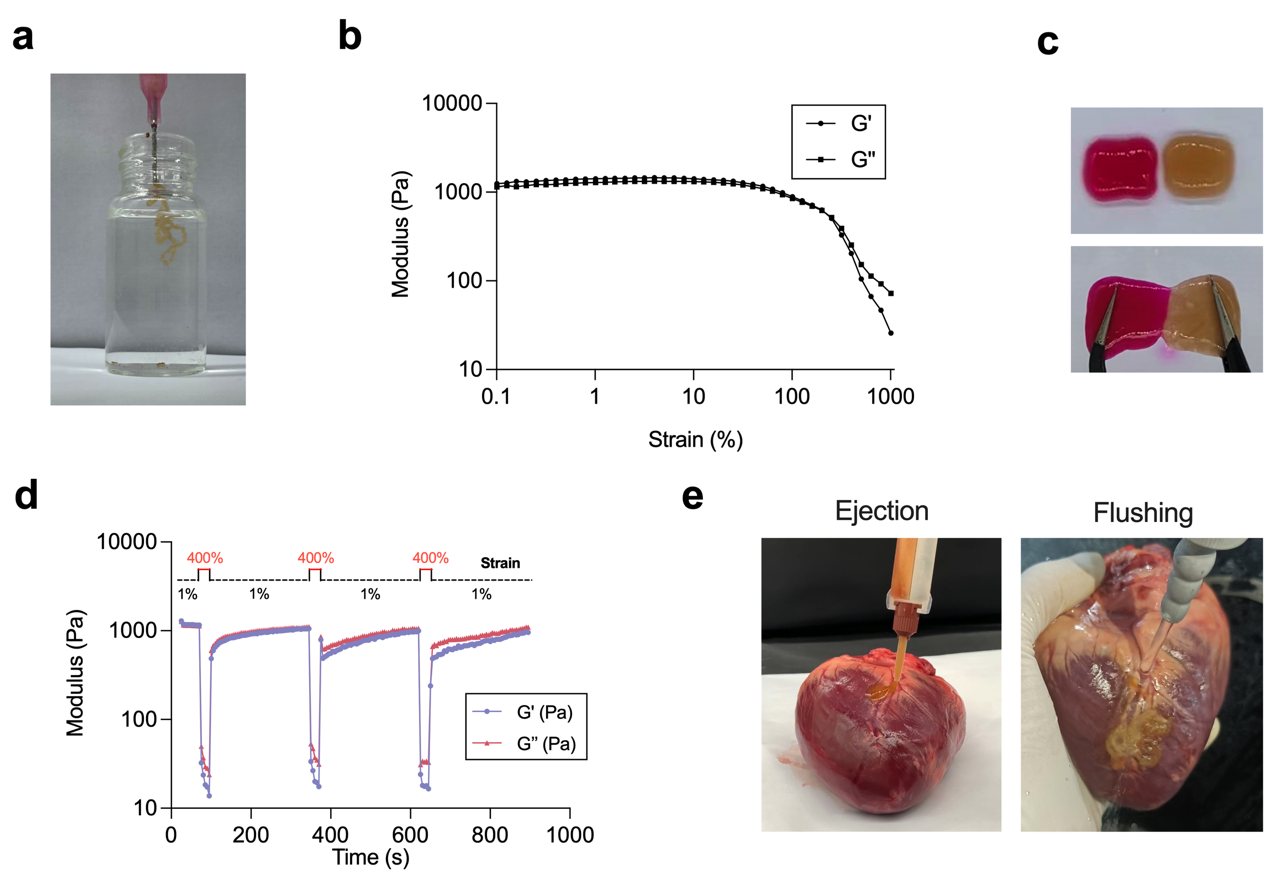


**Supplementary Figure S4 | Injectability, shear thinning and self-healing of VGtn-E.**

**a,** Injection of VGtn-E in water through a 25G needle. **b,** Strain sweep data revealed shear thinning behavior in the VGtn-E hydrogel at 4 Hz. **c,** Self-healing of VGtn-E. **d,** Time sweep data revealed self-recovery of VGtn-E. A strain above the limit for linear viscoelastic responses (400%) was applied to break the hydrogel, followed by a small strain applied to enable the hydrogel to recover. **c,** Ejection of hydrogel precursors using a double syringe with a static mixer.


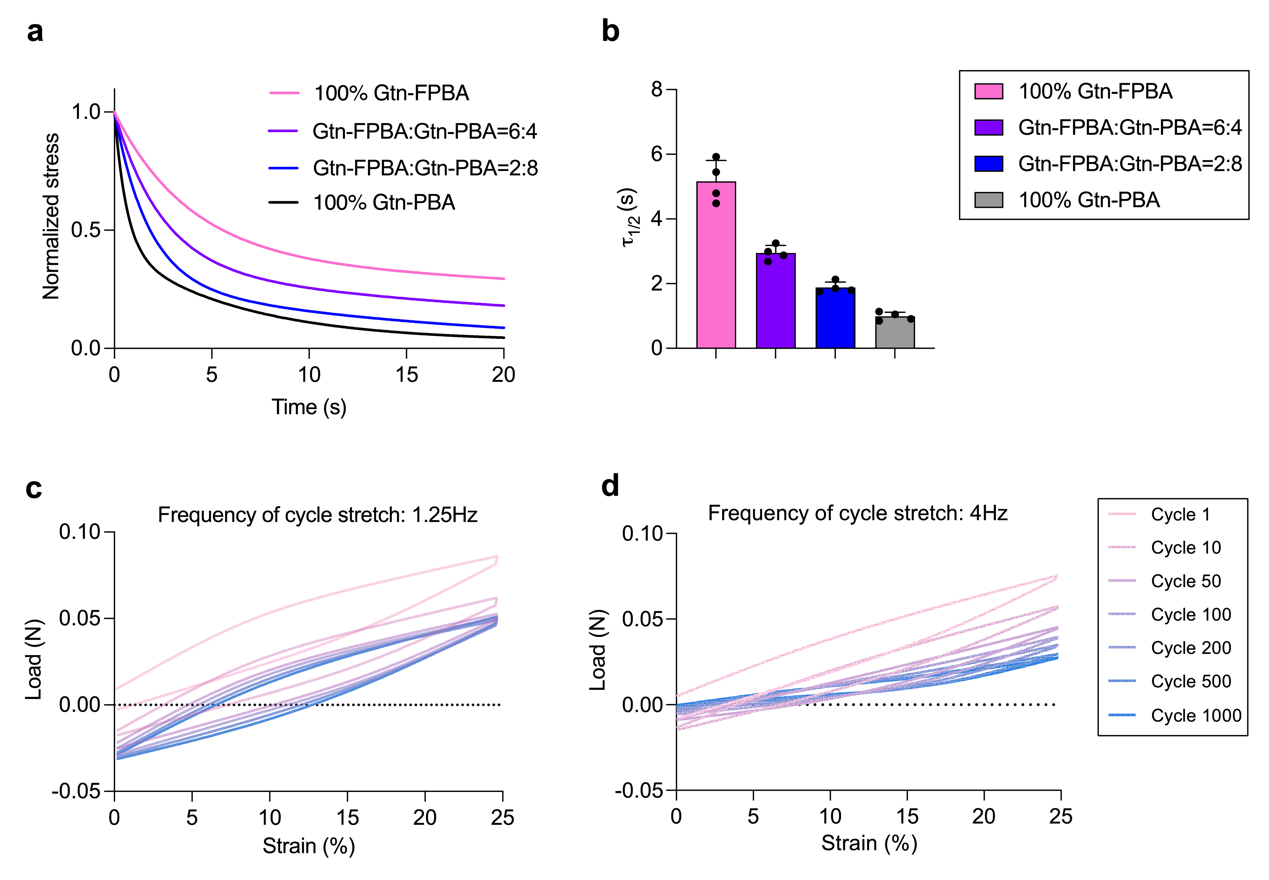


**Supplementary Figure S5 | Stress relaxation and prestress convergence of the VGtn hydrogels.**

**a,** Representative normalized stress relaxation curves of 15% VGtn-E hydrogels. **b,** Statistics of characteristic stress relaxation time of different hydrogels. *n*=5 independent samples for each group. Data are shown as mean ± s.d. and compared by one-way ANOVA followed by Bonferroni’s post hoc test. There were significant differences among all groups (*P* < 0.05). **c** and **d**, Cyclic stretch experiments verify mechanical responses of the VGtn-E hydrogel at 1.25 Hz (mimicking human heart rate) and 4 Hz (mimicking rat heart rate). Load–strain curves reveal prestress convergence of the VGtn-E hydrogel with a pre-strain of 75%. Due to the presence of prestress, the curve does not start from the origin of the coordinate system.


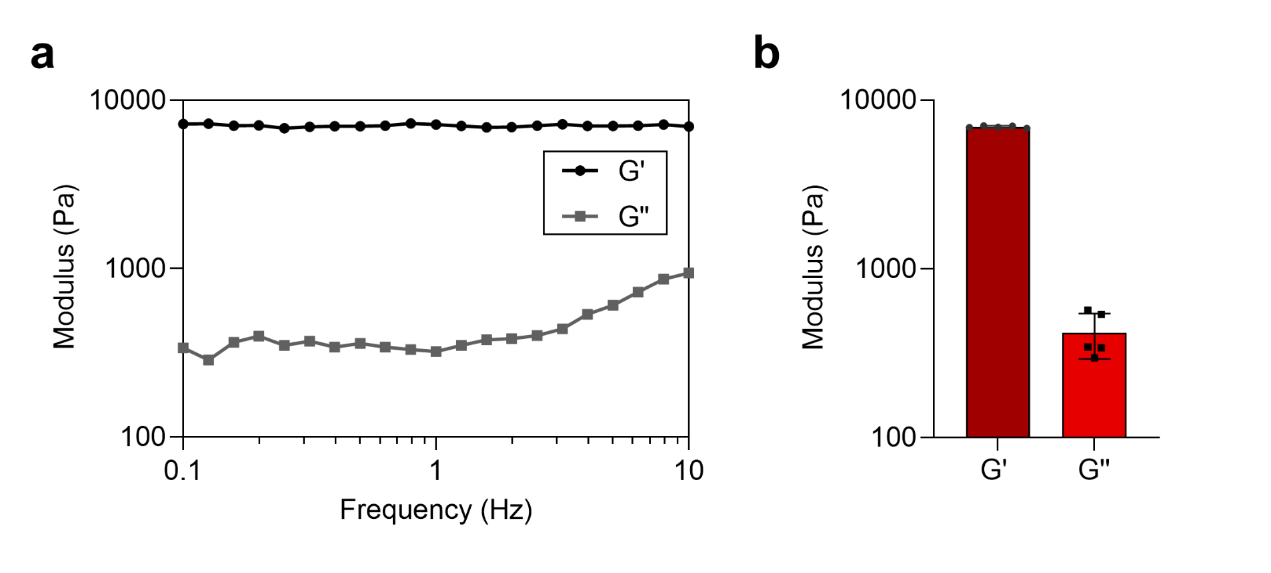


**Supplementary Figure S6 | Mechanical tests of EGtn.**

**a,** Representative rheology curve of EGtn hydrogels. **b,** Modulus of EGtn at 4 Hz, *n*=5 independent samples for each group. Data are shown as mean ± s.d.


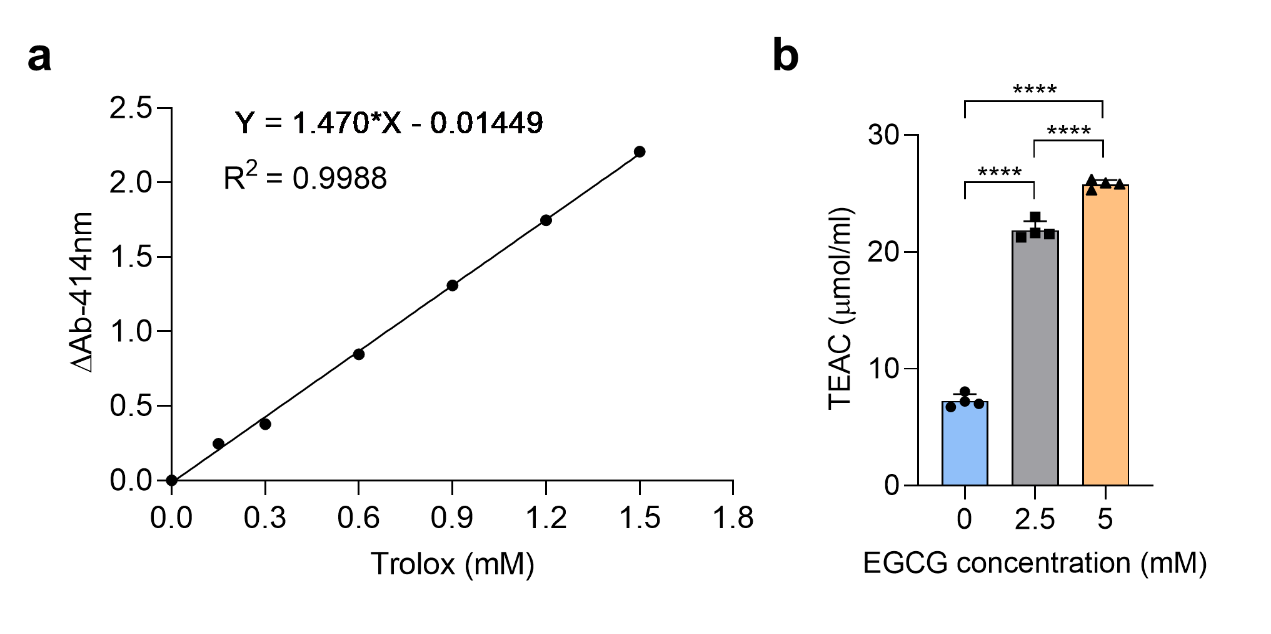


**Supplementary Figure S7 | Antioxidant efficiency of VGtn-E.**

**a,** Absorbance calibration curve relative to Trolox. **b,** Trolox-Equivalent Antioxidant Capacity (TEAC) of VGtn-E with different EGCG concentrations. *n*=4 independent samples for each group. Data are shown as mean ± s.d. and compared by one-way ANOVA followed by Bonferroni’s post hoc test. **** indicates *P* < 0.0001.


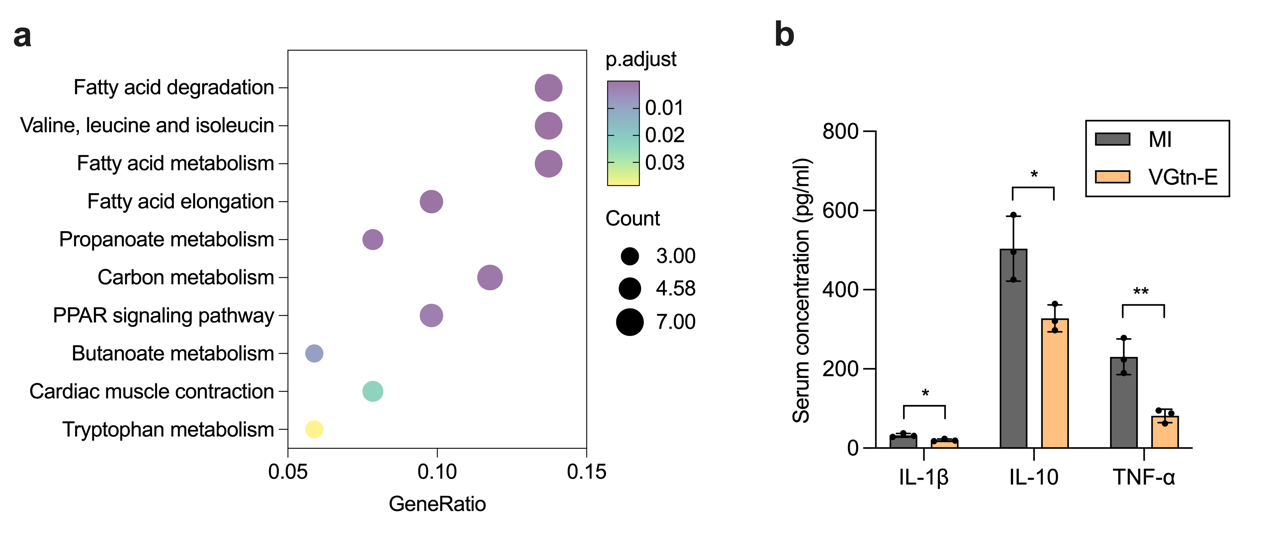


**Supplementary Figure S8 | VGtn-E reduces initial CMs apoptosis and inflammation after MI.**

**a,** Dot-plot of possible differential expression pathways obtained by KEGG enrichment analysis. **b,** Serum concentration of IL-1β, IL-10, and TNF-α detected by ELISA. n = 3 independent samples for each group. Data are shown as mean ± s.d. and compared using a two-tailed Student’s t-test. * and ** indicate *P* < 0.05 and *P* < 0.01.


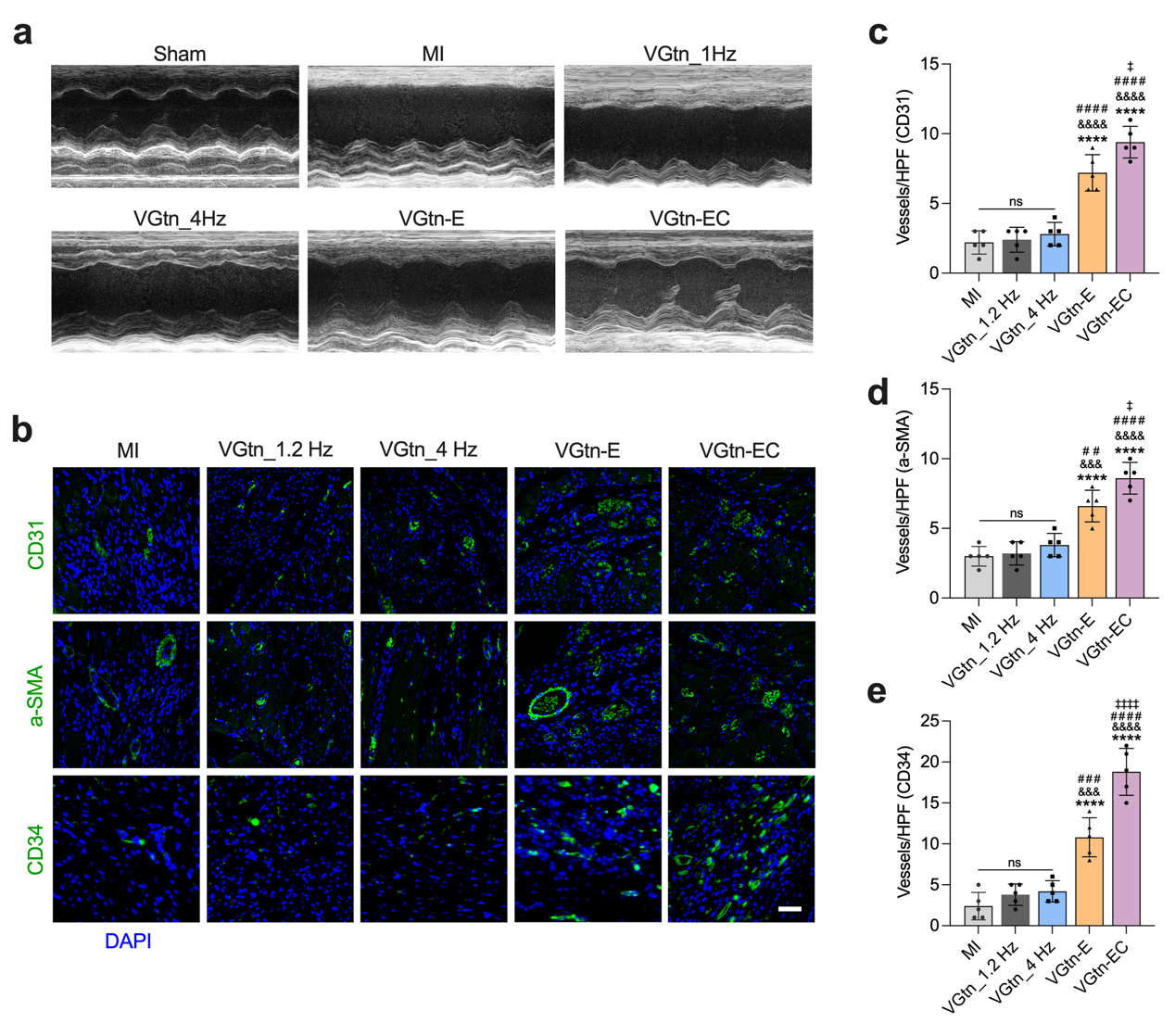


**Supplementary Figure S9 | VGtn improves angiogenesis after MI in a rat model.**

**a,** Representative M-mode echocardiography images of rats with different treatments. **b,** Fluorescent staining of heart section (MI, VGtn_1Hz, VGtn_4Hz, VGtn-E and VGtn-EC) with CD31 (green, top), α-SMA (green, middle), CD34 (green, bottle) and DAPI (blue) to visualize arteries on Day 28. Scale bar, 50 µm. **c**, **d** and **e**, Quantification of the number of arteries in the high-power field (HPF). Data are shown as mean ± s.d. and compared using one-way ANOVA followed by Bonferroni’s post hoc test. ^****^ *P* < 0.0001 compared to the MI group; ^&&&^ *P* < 0.001 and ^&&&&^ *P* < 0.0001 compared to the VGtn_1Hz group; ^##^ *P* < 0.01, ^###^ *P* < 0.001 and ^####^ *P* < 0.0001 compared to the VGtn_4Hz group; ^‡^ *P* < 0.05 and ^‡‡‡‡^ *P* < 0.0001 compared to the VGtn-E group.


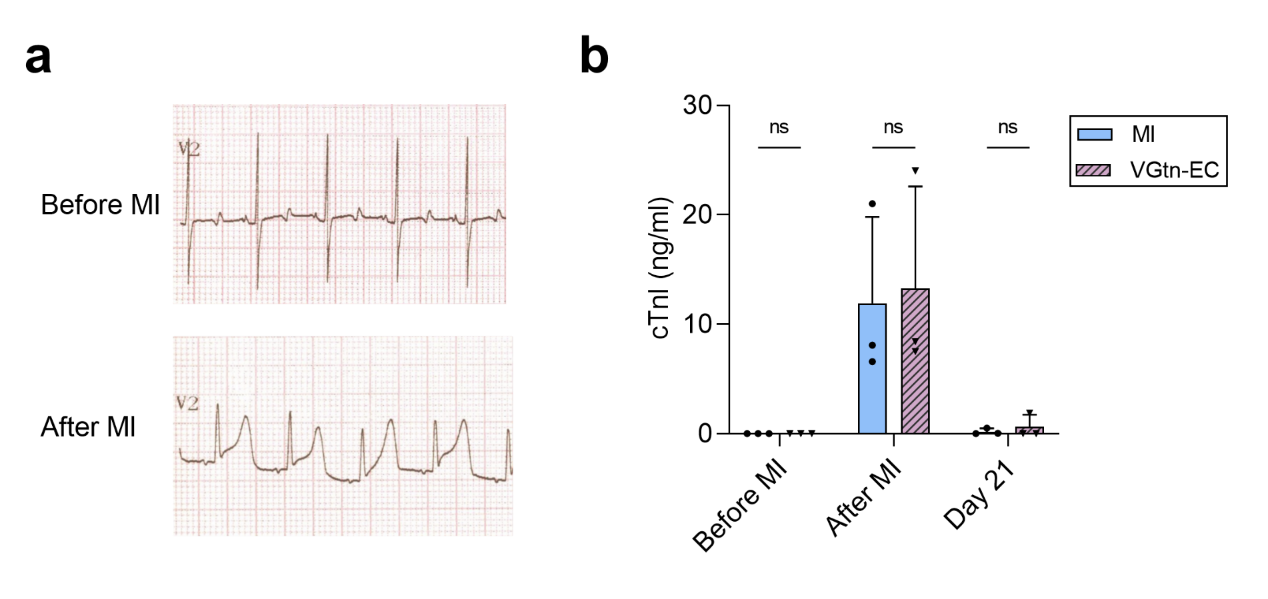


**Supplementary Figure S10 | Evaluation of porcine MI model.**

**a,** Representative electrocardiogram images before ligation and after ligation. **b,** Serum concentration of cardiac troponin I (cTnI). *n*=3 independent samples for each group. Data are shown as mean ± s.d. and compared using a two-tailed Student’s *t*-test.


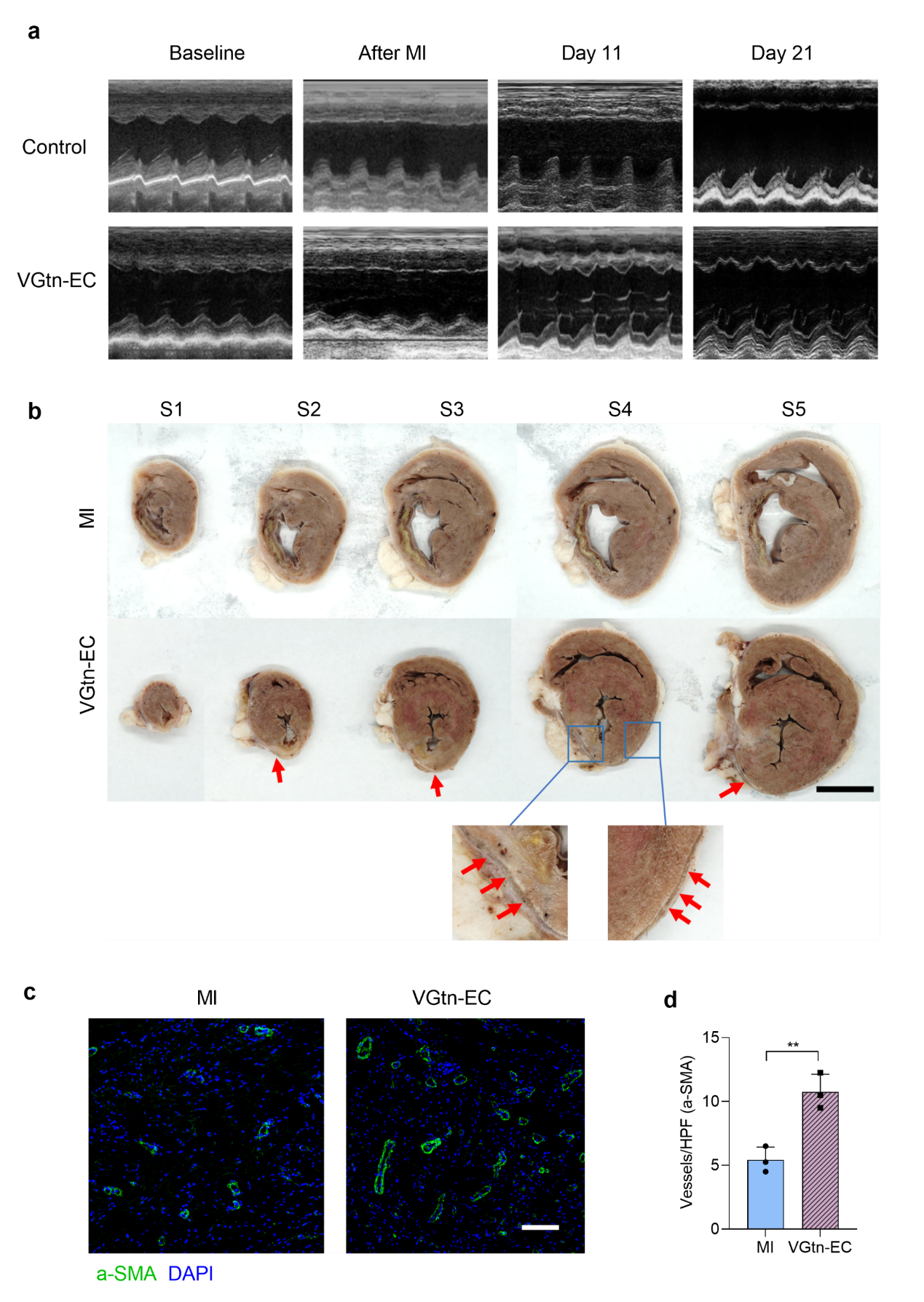


**Supplementary Figure S11 | Improvement of LV function and vascular regeneration after VGtn-EC treatment in porcine MI model.**

**a,** Representative M-mode echocardiography images of MI pigs and VGtn-EC treated pigs. **b,** Representative tissue sections of MI hearts and VGtn-EC treated hearts. The red arrow pointed out the dark brown VGtn-EC between the pericardium and the epicardium. Scale bar, 2cm. **c,** Fluorescent staining of heart section (MI and VGtn-EC) with α-SMA (green) and DAPI (blue) to visualize arteries on Day 21. Scale bar, 50 μm. **d**, Quantification of the number of arteries in high-power field (HPF) form **c**. Data are shown as mean ± s.d. and compared using a two-tailed Student’s *t*-test. ** indicates *P* < 0.01.


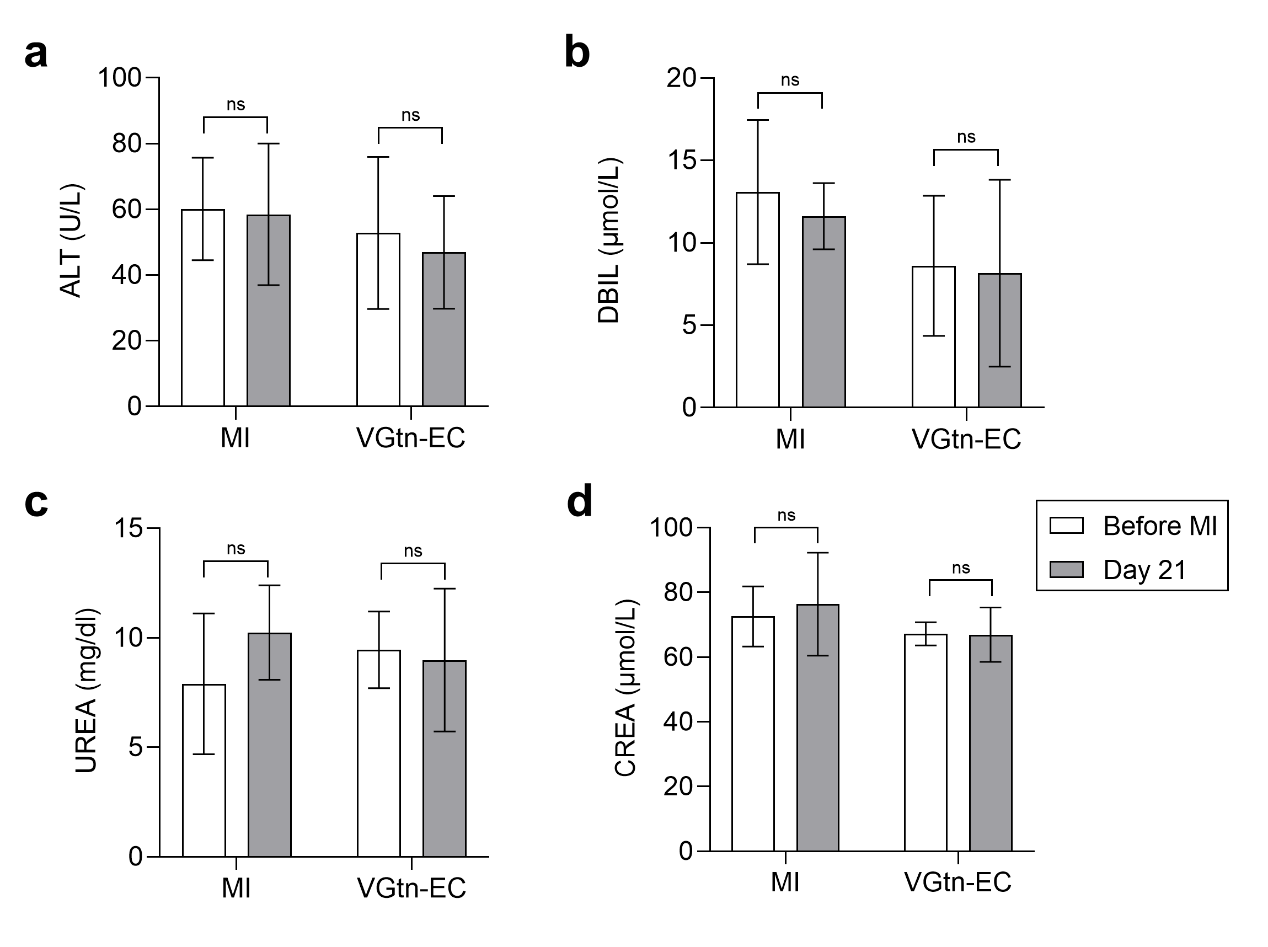


**Supplementary Figure S12 | Effects of VGtn-EC on liver and kidney functions in a porcine MI model.**

**a-d,** Serum concentrations of ALT, BDIL, UREA and CREA of pigs showed VGtn-EC had no liver toxicity or kidney toxicity after implantation. *n*=3 independent samples for each group. Data are shown as mean ± s.d. and compared using a two-tailed Student’s *t*-test.
